# The cross-sectional association between snacking behaviour and measures of adiposity: the Fenland Study, UK

**DOI:** 10.1017/S000711451500269X

**Published:** 2015-09-07

**Authors:** Laura O’Connor, Soren Brage, Simon J. Griffin, Nicholas J. Wareham, Nita G. Forouhi

**Affiliations:** 1 MRC Epidemiology Unit, School of Clinical Medicine, Institute of Metabolic Science, University of Cambridge, Cambridge Biomedical Campus, Cambridge CB2 0QQ, UK; 2 Primary Care Unit, Department of Public Health and Primary Care, School of Clinical Medicine, Institute of Public Health, University of Cambridge, Cambridge Biomedical Campus, Cambridge CB2 0QQ, UK

**Keywords:** Body composition, Adiposity, Obesity, Snacking, Eating patterns

## Abstract

Unhealthy dietary behaviours may contribute to obesity along with energy imbalance. Both positive and null associations of snacking and BMI have been reported, but the association between snacking and total adiposity or pattern of fat deposition remains unevaluated. The objective of this study was to investigate the associations between snacking frequency and detailed adiposity measurements. A total of 10 092 adults residing in Cambridgeshire, England, self-completed eating pattern snacking frequency, FFQ and physical activity questionnaires. Measurements included anthropometry, body composition using dual-energy X-ray absorptiometry scan and ultrasound and assessment of physical activity energy expenditure using heart rate and movement sensing. Linear regression analyses were conducted adjusted for age, socio-demographics, dietary quality, energy intake, PAEE and screen time by sex and BMI status. Among normal-weight individuals (BMI<25 kg/m^2^), each additional snack was inversely associated with obesity measures: lower total body fat in men and women (−0·41 (95 % CI −0·74, −0·07) %, −0·41 (−0·67, −0·15) %, respectively) and waist circumference (−0·52 (−0·90, −0·14) cm) in men. In contrast, among the overweight/obese (BMI≥25 kg/m^2^), there were positive associations: higher waist circumference (0·80 (0·34, 0·28) cm) and subcutaneous fat (0·06 (0·01, 0·110) cm) in women and waist circumference (0·37 (0·00, 0·73) cm) in men. Comparing intakes of snack-type foods showed that participants with BMI≥25 kg/m^2^ had higher intakes of crisps, sweets, chocolates and ice-creams and lower intakes of yoghurt and nuts compared with normal-weight participants. Adjusting for these foods in a model that included a BMI–snacking interaction term attenuated all the associations to null. Snacking frequency may be associated with higher or lower adiposity, with the direction of association being differential by BMI status and dependent on snack food choice. Improving snack choices could contribute to anti-obesity public health interventions.

Eating patterns may contribute to the obesity epidemic along with the effects of energy imbalance. In particular, a shift in eating patterns away from the three-meals-a-day model and towards more of a ‘grazing pattern’^(^ ^1^ ^)^ and an increase in snacking in recent years^(^ ^2^ ^)^ have been proposed to promote obesity^(^ ^3^ ^)^. The prevalence of snacking among adults in the USA increased from 71 to 97 % between 1997/1998 and 2003/2006, and the contribution of snacks to energy intake increased from 18 to 24 %^(^ ^1^ ^)^. Equivalent information on adults in the UK has not been published thus far.

Early investigations often reported an inverse association between eating frequency and adiposity, which was later attributed to reporting bias and the effects of *post hoc* changes in dietary patterns as a consequence of weight gain^(^ ^4^ ^)^. However, snacking frequency may nonetheless be associated with total adiposity or a pattern of fat deposition, as thus far inconsistent findings, both positive^(^ ^5^ ^,^ ^6^ ^)^ and null^(^ ^7^ ^,^ ^8^ ^)^, for an association between snacking frequency and BMI, as the only measure of adiposity, have been reported in adults.

Investigating snacking behaviour has many challenges. First, there is lack of an agreed definition of which meals constitute a snack. Second, there is ambiguity in the terminology as snacking frequency has not been distinguished from consumption of snack foods. Third, snacking frequency has been associated with increases in both ‘healthy’ and ‘unhealthy’ food choices^(^ ^9^ ^)^. Furthermore, physical activity (PA) may confound the associations between snacking and adiposity – for example, sedentary behaviour has been positively associated with consumption of energy-dense snacks and inversely associated with fruit and vegetable consumption^(^ ^10^ ^)^, both of which may be consumed as snacks.

The aim of this study was to investigate the association between self-defined snacking frequency and adiposity measures among adult men and women.

## Methods

### Study population

The Fenland Study is an on-going population-based study designed to investigate the interactions between genetic and lifestyle factors on the risk of obesity and related metabolic traits. Volunteers born between 1950 and 1975 were recruited from general practice lists in and around Cambridgeshire in the East of England. Recruitment began in 2005, and data were available on 10 452 participants at the time of analyses, with a response rate of 27 %.

Exclusion criteria included prevalent diabetes, pregnancy or lactation, inability to walk unaided, psychosis or terminal illness. All volunteers gave their written informed consent, and the study was approved by the Cambridge Local Research Ethics Committee.

Participants were invited to attend one of the three testing sites (Ely, Wisbech or Cambridge) for a single visit, where on the test day they completed a number of questionnaires and assessments as detailed below.

### Exposure: snacking frequency

Participants completed an eating pattern questionnaire in which they were asked to describe the meals or snacks that they usually eat during a 24-h period, using a grid that was divided into 2-h slots and offered four choices of meal type: main meal (e.g. meat with potatoes, pizza, lasagne, fish and chips, burgers, fried breakfast), light meal (e.g. porridge, cereal, toast, sandwiches, soup, salad, omelette), snack (e.g. biscuits, cake, fruit, sweets, chocolate, crisps, nuts, ice-cream) and drink-only snack (e.g. drinks with some milk or sugar in; not ‘low-calorie’ drinks or water). No instruction was given as to whether the 24-h period should be a weekday or a weekend day. Participants were given the option of choosing more than one meal type per time slot. Snacking frequency was calculated as the frequency of food-only snacks that the participant reported. Drink-only snacks were not included due to suggested differences in the satiating effects of foods and drinks^(^ ^11^ ^)^. The analyses were adjusted for frequency of consumption of other meal types to assess the independent association of snacking frequency and measures of adiposity.

### Outcomes: anthropometric and body composition measurements

At the test site facility, weight and height were measured barefoot and wearing light clothing using standardised procedures. BMI was calculated as weight (kg) divided by the square of height (m^2^). Waist circumference was measured mid-point between the lowest rib margin and the iliac crest to the nearest 0·1 cm with a non-stretchable, fibre-glass insertion tape (D-loop tape; Chasmors Ltd).

Body composition was assessed using dual-energy X-ray absorptiometry (DEXA; Lunar Prodigy Advanced fan beam scanner (GE Healthcare)) and ultrasonography (LOGIQ e ultrasound system (GE Healthcare)), and has been described in detail elsewhere.^(^ ^12^ ^,^ ^13^ ^)^ Percentage total body fat was estimated from DEXA using a three-compartment model (fat mass, fat-free mass and bone mineral mass). Visceral and subcutaneous abdominal fat thicknesses (cm) were determined using ultrasound.

### Covariates

Demographic and lifestyle variables were collected using a self-administered questionnaire.

### Dietary covariates

Self-reported habitual dietary intakes over the previous year were estimated using a validated 130-item semi-quantitative FFQ^(^ ^14^ ^)^. Participants were asked to report the frequency of consumption of a ‘medium serving’ on a nine-point scale from ‘never or once per month’ to ‘more than six times per d’ and were asked to complete supplementary questions on milk, breakfast cereal and the type of fat used for baking and frying. Food intake frequency was converted to food (g/d), energy and nutrient intakes using the FFQ EPIC Tool for Analysis^(^ ^15^ ^)^. Foods that could conceivably be consumed as snacks (snack-type foods) were identified from the FFQ and it included fruits, vegetables, yoghurts, nuts, crisps, cakes, biscuits, chocolate, ice-cream and sweets. These were included as covariates in an attempt to rule out confounding by food type in the association between snacking frequency and adiposity.

Plasma vitamin C (µmol/l), an objective marker of fruit and vegetable intake^(^ ^16^ ^)^, was used here as an indicator of dietary quality in line with the inclusion of promoting fruit and vegetable consumption in food-based dietary guidelines^(^ ^17^ ^,^ ^18^ ^)^. Plasma vitamin C levels were assessed from fasting blood samples collected into heparin-containing tubes and stabilised with metaphosphoric acid (10 %) and measured by fluorometric assay within 2 months.

### Physical activity and sedentary behaviour covariates

PA was objectively assessed over 6 d using a combined heart rate and movement sensor^(^ ^19^ ^)^, with individual calibration of heart rate performed using a treadmill test^(^ ^20^ ^)^. Data from free-living were pre-processed^(^ ^21^ ^)^ and modelled using a branched equation framework^(^ ^22^ ^)^ to estimate intensity time-series, which were summarised over time as daily Physical physical activity energy expenditure (PAEE) (kJ/kg per d).

A validated questionnaire, the Recent Physical Activity Questionnaire^(^ ^23^ ^)^, was used to assess usual total PA in the previous 4 weeks. From this, we assessed screen time (hours of TV or video watched per day) as a measure of sedentariness.

### Statistical analysis

After the exclusion of participants who had not completed the 24-h eating pattern questionnaire (*n* 339) and those who reported no usual eating occasions (*n* 21), data from 10 092 participants remained available for analysis. Participants with missing data were retained for analyses; missing categorical data (marital status, *n* 2826; intentional dieting, *n* 2500) were coded as missing, and for missing continuous data (alcohol consumption, *n* 175; years of education, *n* 210) the cohort median value was applied.

Analyses were performed using Stata (version 13; Stata Corp.).

Population characteristics by snacking frequency are presented as mean values (standard deviations) or medians (interquartile ranges) for continuous variables and as numbers (percentages) for categorical variables, and differences by frequency of snacking were examined using ANOVA or a Kruskal–Wallis test for difference or the *χ* ^2^ test for heterogeneity.

Associations between snacking frequency (per unit difference) and body composition were examined using multiple linear regression models. Analyses stratified by sex were conducted *a priori* because previous literature suggested different snacking behaviours by sex^(^ ^9^ ^,^ ^24^ ^)^ and current analyses showing significant interaction between sex and snacking frequency on body composition parameters (*P*<0·05). A pragmatic approach was used to account for potential confounders including demographic, lifestyle, social, dietary and PA factors. Model 1 was adjusted for age (years), smoking status (never, former and current), alcohol intake (units/d), age at completion of full-time education (years), test site (Cambridge, Ely, Wisbech) and other eating occasions (frequency/d); model 2 was further adjusted for energy intake (MJ/d), plasma vitamin C (µmol/l) as a marker of fruit and vegetable intake providing an objectively measured indication of dietary quality, PAEE (kJ/kg per d) and screen time. Occupational social class (routine/manual, intermediate/higher managerial and administrative/professional occupations), marital status (single, married, widowed, separated or divorced) and household income level (<£20 000, £20 000–£40 000 or >£40 000) were also considered but were not significant when entered into the model.

*A priori*, we tested for interaction between snacking frequency and each of BMI (<25 or ≥25 kg/m^2^) and PAEE (< or ≥median: 51·7 kJ/kg per d), in relation to body composition measures.

### Sensitivity analyses

BMR was estimated using Schofield’s equations^(^ ^25^ ^)^. The ratio of energy intake to BMR (EI:BMR) was calculated for each individual. Those with an EI:BMR ratio of <1·14 were classified as under-reporters of energy intake according to cut-off limits developed by Goldberg *et al.* ^(^ ^26^ ^)^. Regression analyses were repeated excluding those who under-reported energy intake.

To investigate the potential effect of intentional dieting on associations with snacking frequency, we compared findings of the analysis of participants who self-reported being on a weight-loss diet with those who reported not being on a diet.

## Results

### Characteristics

Among all, 84 % of the women and 75 % of the men reported snacking at least once per day (range=0–12 times/d). Women reported higher snacking frequency than men (Table 1). Individuals with higher snacking frequency exhibited greater total eating occasions, frequency of other eating occasions and were younger and had higher PAEE compared with their lower snacking frequency counterparts. Higher snacking frequency was associated with lower social class, shorter education duration, lower alcohol intake and being an ex-smoker.

**Table 1:** Characteristics of participants by frequency of snacking*: the Fenland Study, UK (*n* 10 092) (Mean values and standard deviations; medians and interquartile ranges (IQR); numbers and percentages)

Snacking (frequency/d…)	0 (*n* 2040)		1 (*n* 3921)		2 (*n* 2710)		3 (*n* 1047)		4+ (*n* 374)		
	Mean	sd	Mean	sd	Mean	sd	Mean	sd	Mean	sd	*P* †
Age (years)	49·5	7·2	48·2	7·4	46·9	7·3	46·2	7·2	46·0	7·0	<0·001
Main meal (frequency/d)	1·1	0·4	1·1	0·3	1·1	0·4	1·1	0·4	1·2	0·7	<0·001
Light meal (frequency/d)	1·7	0·7	1·6	0·6	1·7	0·7	1·8	0·6	1·9	0·9	<0·001
Energy-containing drink-only snack (frequency/d)	2·9	2·3	3·0	2·3	3·1	2·3	3·2	2·4	3·7	2·9	<0·001
Total eating occasions (frequency/d)‡	5·7	2·3	6·7	2·3	8·0	2·4	9·1	2·5	11·3	3·7	<0·001
BMI (kg/m^2^)	26·9	4·5	26·9	4·7	26·8	5·0	26·7	5·1	26·7	5·1	0·650
Waist circumference (cm)	92·2	13·0	91·2	13·4	89·9	13·7	89·5	13·3	90·1	13·7	<0·001
Total body fat (%)	32·1	8·8	32·9	8·9	33·6	9·2	33·4	9·6	32·7	10·2	<0·001
Visceral fat thickness (cm)	5·5	2·2	5·4	2·2	5·1	2·2	5·0	2·0	5·2	2·1	<0·001
Subcutaneous abdominal fat thickness (cm)	2·8	1·2	2·9	1·2	2·9	1·2	2·9	1·2	2·9	1·3	0·002
Physical activity energy expenditure (kJ/kg per d)	53·1	22·1	53·9	22·3	55·3	21·9	56·3	23·5	57·1	23·4	<0·001
Age at end of full-time education (years)§	18·8	4·6	18·6	4·4	18·8	4·3	19·0	4·4	18·6	4·4	0·022
	Median	IQR	Median	IQR	Median	IQR	Median	IQR	Median	IQR	
Alcohol consumption (units/d)§	1·0	0·4, 2·1	0·9	0·4, 1·7	0·7	0·3, 1·4	0·7	0·3, 1·4	0·6	0·3, 1·3	<0·001
	*n*	%	*n*	%	*n*	%	*n*	%	*n*	%	
Women (%)	856	42·0	2022	51·6	1681	62·0	658	62·9	229	61·2	<0·001
Marital status§											0·159
Single	148	9·9	285	10	170	8·8	71	9·8	26	9·7	
Married	1217	81·4	2322	81·4	1548	80·3	572	79·2	220	82·4	
Widowed/separated/divorced	130	8·7	247	8·7	210	10·9	79	10·9	21	7·9	
Smoking status											<0·001
Never	1042	51·6	2085	53·7	1516	56·8	582	56·3	183	49·6	
Ex-smoker	667	33	1226	31·6	872	32·7	360	34·9	137	37·1	
Current	310	15·4	573	14·8	283	10·6	91	8·8	49	13·3	
Under-reporter for EI (EI:BMR<1·4)	1247	61·1	1913	48·8	1060	39·1	335	32	106	28·3	<0·001
Intentional dieting (yes)§	111	7·5	202	7	176	8·4	73	8·9	32	10·8	0·068
Annual household income level											0·083
<£20 000	265	13·5	566	14·9	360	13·6	128	12·5	50	13·7	
£20 000–£40 000	679	34·5	1361	35·8	967	36·6	395	38·7	148	40·8	
>£40 000	1026	52·1	1876	49·3	1314	49·8	498	48·8	165	45·5	
Occupational social class											0·017
Routine and manual occupations	339	17·8	648	17·8	410	16·3	147	15·2	67	19·1	
Intermediate occupations	508	26·6	113	30·5	786	31·3	302	31·2	104	29·6	
Professional occupations	1060	55·6	1884	51·7	1315	52·4	519	53·6	180	51·3	
Test site											0·001
Cambridge	729	35·6	1294	33	918	33·9	347	33·1	138	36·9	
Ely	704	34·5	1475	37·6	1051	38·8	441	42·1	132	35·3	
Wisbech	609	29·9	1152	29·4	741	27·3	259	24·7	104	27·8	

EI, energy intake.

* Snacking frequency was estimated using an eating pattern questionnaire reflecting usual eating habits over a 24-h period (see the ‘Methods’ section).

† ANOVA or a Kruskal–Wallis test for differences by frequency of snacking or *χ* ^2^ test for heterogeneity.

‡ Total eating occasions=main meal+light meal+snack+drink-only snack.

§ Due to some missing data, numbers do not always add to 10 092 participants.

### Snacking frequency and adiposity

In the adjusted analyses, among women, snacking was positively associated with BMI, waist circumference and visceral and subcutaneous abdominal fat thickness, and was inversely associated with body fat percentage in men (Table 2). Further adjustment (model 2) attenuated the association with body fat percentage in men to null.

**Table 2:** The association between snacking frequency* (per unit increase) and measures of adiposity by sex: the Fenland Study, UK (*n* 10 092) (Mean values and standard deviations; *β* coefficients and 95 % confidence intervals from multiple linear regression analysis)

			Model 1†			Model 2‡		
	Mean	sd	*β* Coefficient	Lower 95 % CI	Upper 95 % CI	*β* Coefficient	Lower 95 % CI	Upper 95 % CI
Women								
BMI (kg/m^2^)	26·5	5·3	0·25	0·09	0·41	0·29	0·13	0·44
Waist circumference (cm)	85·5	12·6	0·7	0·32	1·08	0·73	0·4	1·1
Total body fat (%)	37·6	8·1	0·06	−0·19	0·31	0·12	−0·12	0·36
Visceral fat thickness (cm)	4·3	1·8	0·08	0·02	0·13	0·07	0·02	0·13
Subcutaneous abdominal fat thickness (cm)	3·2	1·2	0·04	0·01	0·08	0·05	0·02	0·08
Men								
BMI (kg/m^2^)	27·3	4·1	−0·06	−0·18	0·07	−0·02	−0·15	0·1
Waist circumference (cm)	97·2	11·5	−0·06	−0·4	0·28	0·01	−0·34	0·35
Total body fat (%)	27·6	7·1	−0·26	−0·48	−0·04	−0·1	−0·32	0·11
Visceral fat thickness (cm)	6·3	2·1	0·01	−0·05	0·08	0·01	−0·05	0·08
Subcutaneous abdominal fat thickness (cm)	2·5	1	−0·02	−0·05	0·01	−0·01	−0·05	0·02

* Snacking frequency was estimated using an eating pattern questionnaire reflecting usual eating habits over a 24-h period. Energy intake was estimated using a FFQ (see the ‘Methods’ section). † Model 1 adjusted for age (years), alcohol (units/d), smoking status (current smoker/non-smoker), age at completing full-time education (years), test site (Cambridge, Ely, Wisbech), main meal (frequency/d), light meal (frequency/d) and drink-only snack (frequency/d) ‡ Model 2: model 1+plasma vitamin C (µmol/l), energy intake (MJ/d), physical activity energy expenditure (kJ/kg per d) and screen time (h)

There was no significant interaction between snacking frequency and PAEE (*P*<0·05) on any body composition measure in men or women. There was, however, a significant interaction between snacking frequency and BMI on waist circumference, body fat percentage and subcutaneous abdominal fat in both men and women (all *P*<0·001) and on visceral fat in women only (*P*=0·002).

**Fig. 1.**
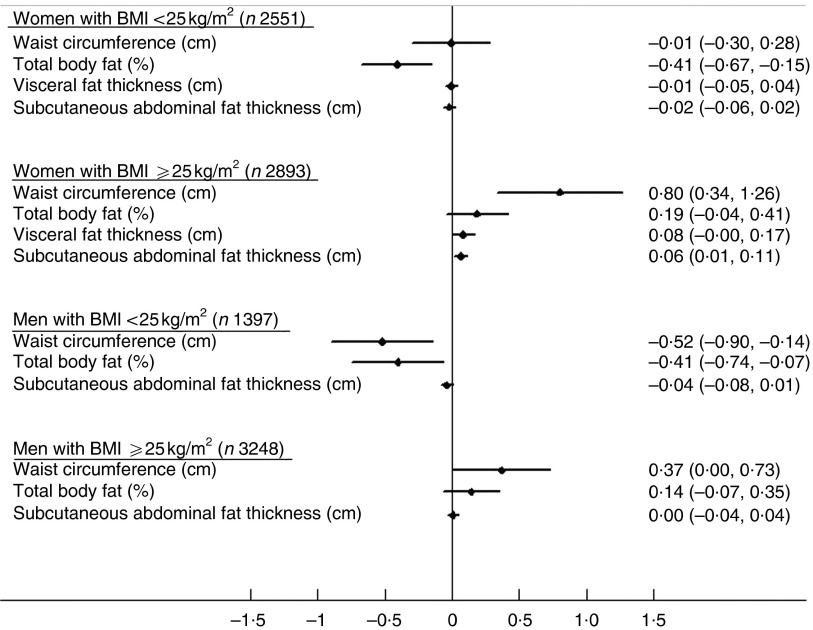
The association between snacking frequency (per unit increase) and measures of adiposity by sex and BMI status: The Fenland Study, UK (*n* 10 092). Data are *β*-coefficients and 95 % confidence intervals from multiple linear regression analysis. Comparison uses model 2, which is adjusted for age (years), alcohol (units/d), smoking status (current smoker/non-smoker), age at completing full-time education (years), test site (Cambridge, Ely, Wisbech), main meal (frequency/d), light meal (frequency/d), drink-only snack (frequency/d), plasma vitamin C (µmol/l), energy intake (MJ/d), physical activity energy expenditure (kJ/kg per d) and screen time (h). No interaction with BMI was noted between snacking and visceral fat thickness in men. Snacking frequency was estimated using an eating pattern questionnaire reflecting usual eating habit over a 24-h period. Energy intake was estimated using a FFQ (see the ‘Methods’ section).

Stratified analysis was conducted where an interaction was significant, using model 2. Among normal-weight individuals (BMI<25 kg/m^2^), there was an inverse association between snacking and body fat percentage in both sexes and between snacking and waist circumference in men (Fig. 1). In contrast, among the overweight or obese subjects (BMI≥25 kg/m^2^), there was a positive association between snacking frequency and waist circumference and subcutaneous fat thickness in women and with waist circumference in men (Fig. 1).

### Snack-type foods

Given the interaction between snacking frequency and BMI, we also examined the differences in the intake of snack-type foods (derived from the FFQ) by BMI status using *t* test or the Mann–Whitney *U* test, as appropriate. Those who were overweight or obese had higher intakes of crisps, chocolates, ice-cream and sweets and lower intakes of yoghurt and nuts compared with their normal-weight counterparts (*P*<0·05) (Table 3).

**Table 3:** Snack-type food intakes* by BMI status: the Fenland Study, UK (*n* 10 092) (Mean values and standard deviations; medians and interquartile ranges (IQR))

	BMI<25 kg/m^2^		BMI≥25 kg/m^2^		
	Mean	sd	Mean	sd	*P* †
Vegetables (g/10 MJ per d)	246·0	248	247·0	203	0·846
Fruit (g/10 MJ per d)	221·0	254	224·0	324	0·573
	Median	IQR	Median	IQR	
Yoghurts (g/10 MJ per d)	25·0	5·3, 66·5	22·3	0·0, 65·1	0·021
Nuts (g/10 MJ per d)	1·7	0·0, 4·4	1·6	0·0, 3·6	<0·001
Crisps (g/10 MJ per d)	2·6	0·6, 10·3	3·3	1·2, 12·4	<0·001
Cakes and biscuits (g/10 MJ per d)	17·0	7·0, 37·0	16·0	6·0, 37·0	0·200
Chocolate (g/10 MJ per d)	4·0	0·8, 10·9	4·5	1·0, 15·9	0·001
Ice-creams (g/10 MJ per d)	3·8	0·0, 6·5	4·2	0·0, 7·4	<0·001
Sweets (g/10 MJ per d)	1·3	0·0, 3·4	1·4	0·0, 3·9	<0·001

* Snack-type food intakes were estimated using a FFQ (see the ‘Methods’ section).

† The *t* test or the Mann–Whitney *U* test for difference.

Moreover, to account for possible confounding by the type of food consumed as a snack, we additionally adjusted the stratified analysis for intakes of snack-type foods. This adjustment did not appreciably change the associations; however, when intakes of snack-type foods were added to the unstratified models, which included a BMI×snacking frequency interaction term, all associations were attenuated to null (results not shown).

### Sensitivity analyses

Exclusion of those categorised as probable energy under-reporters (*n* 4661) did not appreciably change the direction, size or statistical significance of the observed associations, nor did any of the sensitivity analyses. Among those intentionally dieting (12 % of women and 3 % of men), there were no significant associations between snacking frequency and body composition measures, whereas associations among men and women who were not intentionally dieting were similar to those for all men and women (results not shown).

## Discussion

In this large population-based study, we found that snacking frequency was inversely associated with measures of adiposity in normal-weight men and women, but was positively associated among the overweight or obese. Adjustment for overall dietary quality and PA did not affect these associations nor did adjusting for type of snack within the BMI groups. However, differences in the choice of snack between those who were normal weight and those who were overweight or obese informed the opposing direction of association by BMI status.

### Results in context

To the best of our knowledge, this is the first study to examine snacking and adiposity measures other than BMI and to adjust for objectively measured PAEE and objectively measured fruit and vegetable intake as an indicator of dietary quality. Sex differences in the frequency of snacking and in the contribution of snacks to dietary intakes have been reported elsewhere^(^ ^9^ ^,^ ^27^ ^)^. In the present study, a higher frequency of snacking was associated with higher obesity (BMI) and body composition measures (waist circumference, subcutaneous abdominal fat and visceral fat thickness) in women but not among men. These observed sex differences were removed when we stratified by BMI status. After stratification, we found that snacking was inversely associated with total body fat in men and women of normal weight and with waist circumference in men of normal weight, but was positively associated with waist circumference and subcutaneous fat thickness in women who were overweight or obese. No significant associations between snacking frequency and visceral fat were apparent.

Snacking frequency has been associated cross-sectionally with increases in both ‘healthy’ and ‘unhealthy’ food choices, and different dietary patterns have been identified within high-frequency snacking groups^(^ ^9^ ^)^. Other evidences from cross-sectional studies have suggested a modest association between snacking and a more nutrient-dense diet^(^ ^28^ ^)^, and higher intakes of vitamins, carotenoids and minerals^(^ ^29^ ^)^. Snacking is also associated with eating more in general and choosing a wider variety of foods, resulting in a more balanced intake of nutrients^(^ ^30^ ^)^. The categorical term ‘snacks’ includes energy-dense and nutrient-poor foods, which are commonly referred to as snack foods, and also low energy-dense and high-fibre foods such as fruits. It is also generally thought that increased snacking is associated with lower PA levels, but the causal mechanism and the direction of the relationship remain uncertain. Bertéus Forslund *et al*.^(^ ^27^ ^)^ concluded that high PA could not explain high-energy intake and snacking, having found that energy intake was higher with higher snacking frequency, irrespective of PA level, but it has also been suggested that the effects of snacking on weight gain may be mediated by PA through increased energy requirements^(^ ^31^ ^)^. This disparity in the relationship between snacking frequency and dietary intakes and PA has been suggested as masking, and thus preventing the detection of an association between snacking and obesity or weight gain^(^ ^5^ ^–^ ^8^ ^,^ ^30^ ^)^. However, we found that accounting for the interaction with BMI status allowed for the detection of an association. There were different snack-type food intakes between the two groups, with those who were overweight or obese consuming less yoghurt and nuts and more ice-cream, sweets, chocolate and crisps compared with their normal-weight counterparts. One possible explanation for the different associations between BMI groups is differential gut hormone responses that different foods or nutrients may elicit. Gut hormones are known to both curb and increase appetite – for example, ghrelin may increase appetite and oxyntomodulin and peptide YY_3-36_ may increase satiation^(^ ^32^ ^)^. In addition, a previous study has shown that healthy, non-obese adults may maintain a normal body weight by compensating for the consumption of snacks with increased PA or reduced energy intake at other meals^(^ ^33^ ^)^.

### Reporting bias

The potential of intentional dieting contributing to the differential trends by BMI status was discussed at a symposium^(^ ^34^ ^)^ in 2012 and is supported in the present analysis as women with higher BMI reported lower energy intakes. Overall, our findings do not suggest that intentional dieting was driving the associations; however, we acknowledge that the number of individuals on a weight-loss diet in these analyses was small, and thus the effect of snacking among those on a diet may warrant further investigation.

As one of the major causes of dietary under-reporting is the failure to report foods eaten between meals^(^ ^35^ ^)^, reporting bias has the potential to create spurious results in any analysis of snacking. Consistent with this, we found that the percentage of energy under-reporters decreased as reported snacking frequency increased. However, the associations of snacking frequency with BMI and body composition remained similar after excluding potential under-reporters. This is in contrast with the eating frequency research, where the associations were attenuated to null when under-reporting was accounted for^(^ ^4^ ^)^. In addition, it is possible that participants associate snacking with weight gain, which may cause selective under-reporting of snacking frequency by those who are overweight or obese, thus causing the differential associations, although reported snacking frequencies did not differ by BMI status in the present study.

### Strengths and limitations

The Fenland Study has a large population-based sample drawn from Cambridgeshire, which is representative of the general England population in terms of smoking and PA levels and somewhat healthier than the England average for obesity levels and healthy eating^(^ ^36^ ^)^. The strengths of the study are the use of many objective and precise measures of body composition, including DEXA and abdominal ultrasound, the inclusion of plasma vitamin C as an objectively measured marker of fruit and vegetable intake reflecting dietary quality and the inclusion of objectively measured PAEE. Our approach to analysis was rigorous, as we investigated possible under-reporting, intentional dieting and interaction by BMI and PAEE, allowing us to account for and exclude many alternative explanations for an association between snacking frequency and adiposity.

A further strength is that the present analysis may better reflect usual snacking behaviour of participants, capturing what they deem to be snacks. Although often defined as foods eaten between meals, there is no physiological basis to distinguish a meal from a snack^(^ ^37^ ^)^. A biologically based definition has been proposed: eating during a period of satiety rather than simply eating between meals^(^ ^34^ ^)^, while self-definition by participants has also been advocated^(^ ^38^ ^)^. In this study, participants were asked to describe their usual eating patterns for a 24-h period, giving information on the time of the day and the frequency of consumption of each meal type, whereas other studies have recorded snacking during a specific period of time or derived frequencies from food intake records where the researcher decides meal types.

This study has certain limitations. Its cross-sectional nature prevents the direction of the association from being determined, and we could not investigate the association of snacking with weight change. We were unable to validate the eating pattern (snacking frequency) questionnaire or examine the definition of a snack, drawing no distinction between eating between meals or eating in a state of satiety and relying on participants’ interpretation of what constitutes a snack. As eating pattern data (24-h eating pattern questionnaire) were collected separately from dietary intake data (FFQ), the energy and nutrient content of the snacks and which specific foods were eaten as snacks could not be determined. We did, however, make approximations using the FFQ data. We did not include drink-only snacks when estimating snacking frequency, as we considered snacking on foods and drinks to have different satiating effects; however, the analyses were adjusted for drink-only snack frequency. Although our assessment was comprehensive, data on eating patterns and dietary intake were self-reported and are subject to measurement error and bias that accompany such subjective assessment. We adjusted for a range of relevant potential confounding factors but residual confounding could not be ruled out.

### Conclusion

In conclusion, snacking frequency was inversely associated with measures of adiposity in normal-weight men and women, but was positively associated in those who were overweight or obese. The differential association by BMI group may be due to differences in the choice of snack. The promotion of healthy snack choices could contribute to anti-obesity public health initiatives.
